# Investigation of Adhesive Joining Strategies for the Application of a Multi-Material Light Rail Vehicle

**DOI:** 10.3390/ma14226991

**Published:** 2021-11-18

**Authors:** Yiding Liu, Craig Carnegie, Helen Ascroft, Wenhao Li, Xiao Han, Hua Guo, Darren J. Hughes

**Affiliations:** 1 Warwick Manufacturing Group, University of Warwick, Coventry CV4 7AL, UK; craig.carnegie@warwick.ac.uk (C.C.); helen.ascroft@warwick.ac.uk (H.A.); d.hughes@warwick.ac.uk (D.J.H.); 2 Centre of Excellence for Aeronautics, School of Aerospace, Transport and Manufacturing, Cranfield University, Cranfield, Bedfordshire MK43 0AL, UK; wenhao.li@cranfield.ac.uk; 3 Department of Engineering Mechanics, Dalian University of Technology, Dalian 116024, China; hanxiao@dlut.edu.cn; 4 Research Institute for Clean Growth and Future Mobility (CGFM), Coventry University, Coventry CV1 5FB, UK; hua.guo@coventry.ac.uk

**Keywords:** multi-material light rail vehicle, adhesive bonded joints, finite element model, single lap joint, variation in bond gap

## Abstract

To meet the high demand for lightweight energy-efficient and safe structures for transport applications, a current state-of-the-art light rail vehicle structure is under development that adopts a multi-material design strategy. This strategy creates the need for advanced multi-material joining technologies. The compatibility of the adhesive with a wide range of material types and the possibility of joining multi-material structures is also a key advantage to its success. In this paper, the feasibility of using either epoxy or polyurethane adhesive joining techniques applied to the multi-material vehicle structure is investigated. Importantly, consideration is given to the effect of variation in bond thickness for both families of structural adhesives. Multi-material adhesively bonded single lap joints with different adhesives of controlled bond thicknesses were manufactured and tested in order to experimentally assess the shear strength and stiffness. The torsional stiffness and natural frequency of the vehicle were modelled using a global two-dimensional finite element model (FEM) with different adhesive properties, and the obtained vehicle performances were further explained by the coupon-level experimental tests. The results showed that the vehicle using polyurethane adhesive with a target bond thickness of 1.0 mm allowed for optimal modal frequency and weight reduction.

## 1. Introduction

When compared with the aerospace, marine and automotive industries, the rail industry is generally perceived to have been slow in its adoption of new materials; e.g., composites, in load-bearing structural components. This is generally recognised to be due to the lack of suitable certification procedures, with conventional high-strength steel being most commonly used to construct a vehicle body [1]; however, the requirement for a long-term financial and environmental viability of the rail network has pushed the transportation industry to engage in the application of a lightweight multi-material design strategy for primary structures [2]. This includes integrating a mix of components made from lightweight energy efficient materials, e.g., aluminium alloys and composites. This is also the current state of the art in the automotive sector to use the “right material in the right place”, which is a key driver of the application of adhesives.

The traditional steel structures within rail vehicles are primarily joined using welding techniques, such as spot/arc welding, which are fast, robust, and low-cost [3]. Nevertheless, welding requires high temperatures, resulting in a brittle layer of intermetallic forming at the joint interface in some body metals, as well as high levels of residual stress. This makes it difficult to obtain the desired joint strength, as well as presenting premature failure due to corrosion; which calls for more frequent inspections. The usage of adhesive joining is increasing in current rolling stock (inside wall decorating sheets, floor plates, and floor coverings [4]) due to its compatibility with a wide range of material types and the possibility of combining multi-material structures. Adhesives also exhibit a high strength-to-weight ratio, uniform stress transfer, design flexibility, damage tolerance, and crash/fatigue resistance over conventional joining methods, as well as acting as a water ingress barrier [5].

There are still factors, however, that limit both the optimum design and confidence in adhesives, such as joint geometry, adhesive types, and thermal mismatch stresses. To date, a comprehensive number of works and review papers [5,6,7,8] have been undertaken to study the performance of adhesive-bonded joints. Specific studies focused on geometric parameters [9,10,11,12,13] (adhesive thickness, overlap length, joint configuration), material parameters [14,15,16,17] (adhesive and adherend materials), and surface conditions. It has been shown that a greater bonding interface is created once surface preparations such as air blasting, abrasive paper marking, plasma, and liquid cleaning agents have been utilised. All of these improve surface properties, including wettability, energy, and contact angle [18]. Increasing said energy enhances the interlock effect between adherends and adhesives, improving the overall bond [19]. Another key parameter that influences the mechanical performances of bonded joints is the adhesive thickness. Da silva et al. [20] carried out tests on steel single lap joints (SLJs) bonded with different adhesive types (from brittle to ductile adhesives) and different adhesive thicknesses (0.2, 0.5 and 1.0 mm). The results suggested that the lap shear strength increased as the bondline reduced and the adhesive property becomes stiffer. Arenas et al. [21] investigated the thickness effect of an acrylic adhesive on the lap-shear strength of aluminium SLJs. The results suggested using a thickness between 0.4 and 0.8 mm to target a cohesive failure range. Banea et al. [22] studied the influence of adhesive thickness on the mechanical behavior of a structural polyurethane adhesive. The results illustrated that the ductile adhesives performed better with slightly thicker bond lines. What is largely lacking in the literature are studies relating adhesive selection and thickness to the performance of a full body structure. This paper attempts to fill that gap, specifically for the application to a state-of-the-art light rail vehicle.

**Figure 1:**
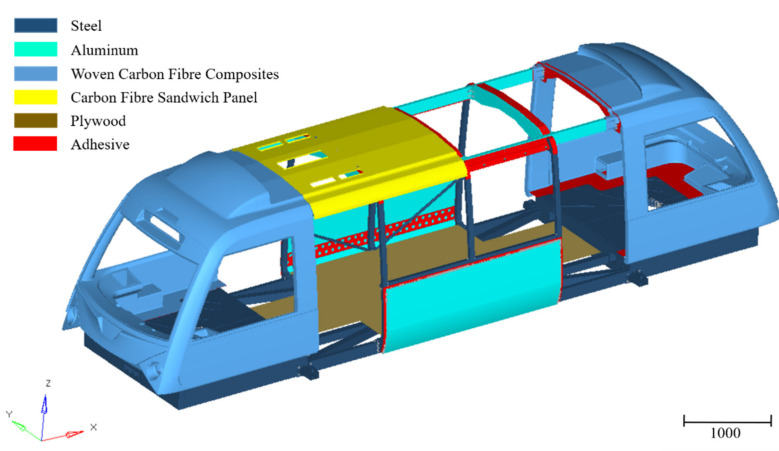
Multi-material design used in a light rail vehicle structure under development. Units are in mm. The vehicle length, width, and height are 11.0, 2.7 and 3.1 m, respectively.

Lightweight design of railway vehicles is applied to conserve energy; however, using light materials and adopting a lightweight structural design may also result in deterioration of rigidity of the vehicle body [23,24]. This reduction could result in low modal frequencies and strong vibrations, impacting vehicle stability and passenger comfort. The use of an adhesive with a high damping capacity can increase the damping of a bonded joint [25]; however, high damping in an adhesive is usually associated with relatively low stiffness and strength. Hence, a balance between vibration amplitude and static strength and stiffness needs to be considered. This paper presents the development and potential application of a light rail vehicle using multi-materials for economic benefit, as shown in Figure 1. For example, the nose of the vehicle is made of woven carbon fibre composites for higher stiffness, and the upper chassis is made of aluminium for weight reduction. Due to the limited load transfer path between dissimilar materials, a large amount of adhesive bonding has been recommended in the main structure assembly (e.g., top chassis to roof assembly; side module structure to roof assembly and skin panel; nose to bogie mount). As various adhesive types and adhesive thicknesses behave differently in structural performance and energy absorption, it is important to select the most suitable adhesives by linking the demanding mechanical requirements and the realistic manufacturing considerations. For example, during vehicle production it is reasonable to expect some degree of dimensional misalignment between panels (during fit-up). In these situations, the adhesives ability to “gap fill” is important. Clearly, an understanding of joint performance with gap magnitude is critical to the designer. In reference [26], Galvez et al. recently analysed the viability of a polyurethane-based adhesive for the connecting joint in a steel bus structure and validated its maximum shear stress in laboratory tests. This is a rare but good example demonstrating the linking of laboratory joint testing to the component performance under realistic design and loading scenarios.

The aim of the present work was to evaluate adhesive types and bond line thicknesses on the torsional stiffness and modal performance of the light rail vehicle structure currently under development. Two different kinds of adhesives (SikaFlex 265 polyurethane adhesive and DP490 epoxy adhesive) were considered in this study. Composite-to-aluminium and aluminium-to-aluminium bonded joint coupons with different polyurethane and epoxy adhesive thicknesses were manufactured and tested in the laboratory to evaluate the thickness effect of the joints shear strength and joint stiffness. A two-dimensional global finite element model (FEM) of the entire vehicle was then developed to examine the structural torsional stiffness and free-vibration natural frequency of the global structure by modelling different adhesive scenarios. The mechanical performances of vehicle were explained by the observations from the coupon tests of epoxy and polyurethane adhesives, respectively. Finally, a recommendation was given on the selection of adhesive in improving the structural integrity of the vehicle.

## 2. Single Lap Joint Tests to Characterise Different Adhesives

This study considered the use of two different kinds of adhesives for investigation: SikaFlex 265 adhesive (1-C polyurethane) and DP490 adhesive (two-component epoxy adhesive with base to accelerator mixed at 2:1 by volume). The key reasons to choose these two adhesives were because they are welcomed and easily accessible adhesives in the laboratory selected for the project. The single lap joint configuration test coupons were manufactured, as the configuration represented the simplest geometry shape.

**Figure 2:**
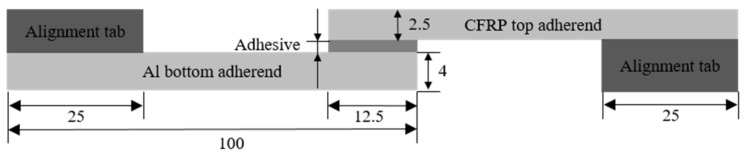
Geometry and dimensions of single lap joint (SLJ) specimens following the guidance of the BS EN 1465:2009 standard. The width of the single lap joint was 25 mm (polyurethane joint design shown; units in mm).

SikaFlex 265 adhesive, provided by Sika Ltd. (Baar, Switzerland), is a high-performance, one-component gap-filling polyurethane (PU) adhesive designed for structural assemblies. Hybrid aluminium-composite bonded joints with different adhesive thicknesses (0.3, 0.5, 1.0, 2.0 and 4.0 mm) were manufactured in this work. The aluminium alloy 6110A-T6 used in this study was introduced in the automotive industry recently. The composites used for the bonding were cut from a high-strength woven carbon fibre reinforced plastic (CFRP) sheet, provided by EasyComposites Ltd. (Stoke-on-Trent, UK) Geometry and characteristic dimensions of the SLJ specimens are shown in Figure 2. The width of the joint was 25 mm. It was necessary to ensure an acceptable and desirable failure of the joints with the adhesives by using an appropriate surface treatment. For both the aluminium and composite substrates, the surfaces were treated by abrasive sanding using P40 sandpaper, followed by a cleaning process with ethanol solvent. A thin layer of Sika Primer 204N was applied to favor the chemical bond at the adherend–adhesive interface. The adhesive thickness was controlled by using glass beads of a given diameter. It was found to be difficult to bond thicker polyurethane adhesive joints (larger than 2 mm), as the polyurethane adhesive had a large viscosity. Therefore, it was suggested in the adhesive datasheet [27] that the adhesive be applied in a triangular shape with the assistance of a designed jig to ensure the proper bonding length. A uniform layer of adhesive was ensured by applying pressure on the bonded region using metal clips. The polyurethane adhesive was allowed to cure at room temperature and left for seven days before testing in line with supplier recommendations.

DP490 epoxy adhesive is a two-component structural adhesive designed for metal and composite assembly supplied by 3M Ltd. (Loughborough, UK) Aluminium-to-aluminium substrates of 2 mm thickness were bonded using the same geometry and dimensions as shown in Figure 2. Aluminium substrates were chosen in order to provide consistent results due to the fact that the CFRP failed in delamination in the initial epoxy trial, and the aluminium material has a similar Young’s modulus to the woven CFRP, thus aluminium substrates were selected to show the effect of the adhesive thicknesses with a similar Young’s modulus to woven laminates. The surface treatment included a grit-blasting abrasive process, followed by cleaning using isopropanol alcohol to provide a clean bonding surface, based on suggestions by 3M Ltd. [28]. The maximum thickness of epoxy bond gap was 2 mm, as suggested by 3M Ltd. [28], therefore the thicknesses selected for the epoxy adhesive were 0.3, 1.0, and 2.0 mm. A thickness of 0.5 mm was not selected due to the fact that the two packages of tests were conducted at different times. The same gap control procedure as used with the polyurethane adhesive was employed. The curing was completed for half an hour at 95 °C, and the samples were returned to room temperature before testing in line with supplier recommendations.

All the lap-shear tests were conducted using a 30 kN universal testing machine with a cross-head speed of 1 mm/min, according to the BS EN 1465:2009 [29] standard. The repeatability of the experiments was optimised with strict control of the environmental temperature and humidity. Three repeat samples of each bonding scenario were tested (for the 4 mm PU adhesive; only two samples were tested due to the difficulty in preparing samples). The loads and extensions were recorded by the test machine.

## 3. Test Results

### 3.1. Load-Displacement Curves

The extension measurements recorded by the cross-head sensor included the machine’s compliance; this was due to the small tolerances that were injected into the machine with multiple moving parts (cross head, grip inserts, grip teeth, lap shear tabs). In order to mitigate this, a correction test was conducted in conjunction with an extensometer, situated on an epoxy single lap joint, across the span of the adhesive.

**Figure 3:**
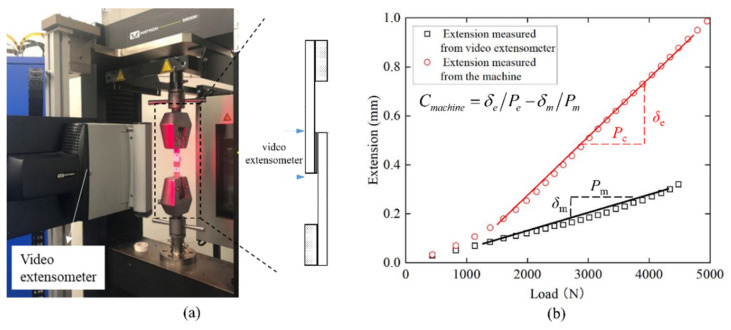
(**a**) Experimental setup with video extensometer; (**b**) extension vs. load relationship measured with video extensometer and machine, respectively. Units are in mm.

The video extensometer was used to measure the relative displacement between the two dots at a very close distance to the adhesive overlap, as shown in Figure 3a. The compliance of the machine was calculated using the compliance with the extension measured from the machine subtracted by the compliance with the extension measured from the video extensometer as shown in Figure 3b, which was 1.47 × 10^−4^ mm/N. The stiffness of joints were calculated as the slope of the load-displacement line in the range from 5% to 10% of the maximum extension plotted in Figure 3 divided by the joint width, as it was considered that during this period the adhesive could be assumed primarily in bearing the shear load if the elastic modulus of substrates materials was large enough compared to the modulus of the adhesive. This measured compliance of the machine was then used to correct the extension of the load-displacement curve; therefore, the extension presented in the following curves are based on the corrected extension.

**Figure 4:**
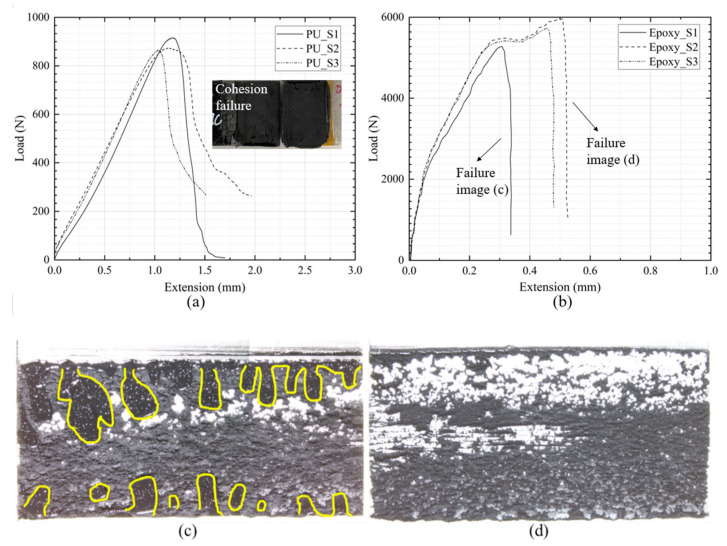
Load-extension curves of the single lap joint specimens with polyurethane adhesive (**a**) and epoxy adhesive (**b**) at 0.3 mm thickness; (**a**) shows the cohesive failure of the test sample. The inset pictures in (**c**,**d**) indicate the fracture surfaces of the specimens obtained from an optical microscope for sample Epoxy_S1 and sample Epoxy_S2; the image associated with sample Epoxy_S1 contains a number of highlighted voids (yellow outline).

Figure 4 shows the comparison of load-extension curves of three tested specimens with (a) polyurethane and (b) epoxy adhesives with a 0.3 mm bondline thickness. The load-extension curves for other bonding scenarios are shown in Appendix A. Joints with 0.3 mm epoxy adhesive could sustain approximately six times the failure load compared to that of the polyurethane adhesive. The extension at failure for the epoxy adhesive joints, however, was of the same order as that of the polyurethane joints. Some variations among the different specimens could be seen for both the epoxy and polyurethane adhesives, which is typical in bonded specimens and cannot be avoided. It was postulated that the lower load capacity, as obtained for specimen Epoxy_S1 with epoxy adhesive, was caused by a reduction in the effective bonded area (the fracture surfaces of the specimen S1 contained a number of voids as a result of the protective coating on the sheet aluminium surface, as highlighted in Figure 4c). The consistency of the load capacity was good for the polyurethane adhesive, as cohesion failure modes were obtained for all specimens. Although the extension at the maximum load had some scatter (11% difference for the epoxy adhesive), the overall trend regarding different adhesive thicknesses was still within an acceptable range.

### 3.2. Failure Strength and Extension of Polyurethane Adhesive Joints with Respect to Bond Gap

**Figure 5:**
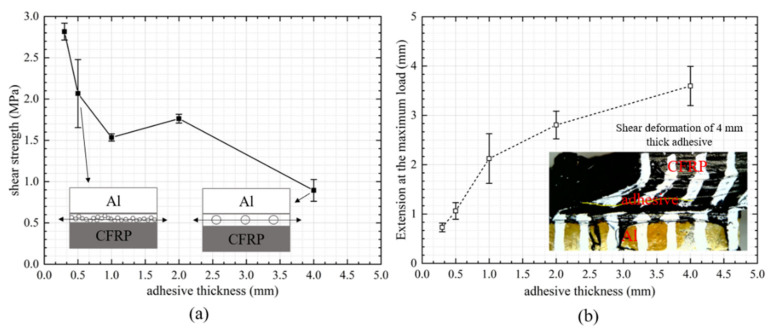
(**a**) Shear strength and (**b**) extension at the maximum load vs. thickness of the polyurethane adhesive. The inset pictures indicate the failure of the polyurethane joints. The figures in (**a**) indicate the failure path in the sample, with the round circles representing the embedded glass bead. The figure in (**b**) shows the shear deformation of the polyurethane joint with the 4 mm thick adhesive.

**Figure 6:**
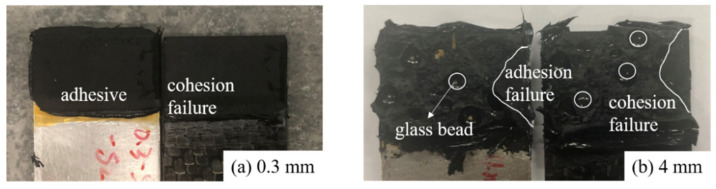
Failure surfaces of joints with polyurethane adhesives with thicknesses of (**a**) 0.3 mm and (**b**) 4 mm. The white region close to the edge for the 4 mm bond is an adhesion interface failure.

Figure 5 summarizes the results of (a) failure strength and (b) extension at the maximum load as a function of adhesive thickness for the polyurethane adhesive used. The failure strengths of the joints were calculated using the failure load divided by the bonded area. For polyurethane adhesive, a general decreasing trend from around 3 MPa to less than 1 MPa was observed with increasing adhesive thicknesses from 0.3 to 4.0 mm, respectively. The polyurethane adhesive exhibited a larger plastic zone due to excessive deformation as the adhesive thickness became thicker, leading to damage and final fracture failure. Conversely, the extension at the failure load increased with the increasing adhesive thicknesses. It was found that the polyurethane adhesive carried a large shear deformation without bending the substrates; e.g., for the 4 mm-thick adhesive, the shear elongation at failure went up to roughly 100%. The failure modes of the bonded joints with polyurethane adhesive thicknesses of (a) 0.3 mm and (b) 4 mm are shown in Figure 6. It is interesting to note that the failure presented different particularities as a function of the adhesive layer thickness. For example, when the adhesive thickness was smaller than 2 mm, fracture occurred in cohesion failure with the composite substrate covered with a very thin layer of adhesive. In contrast, as the adhesive thickness reached 4 mm, the cracks tended to initiate from the middle of the adhesive, resulting in cohesion failure or mixed failure (Figure 6b). Banea et al. [22] found completely different failure particularities in their study of another polyurethane adhesive (the fracture occurred in the middle of the adhesive layer when the adhesive was thin and near the interface when the adhesive was thicker). This phenomenon was explained such that the interface stresses increased as the bondline became thicker, and therefore the failure occurred close to the interface for the thicker adhesive. In this study, however, the glass bead used for thicker adhesive bond was found to significantly alter the crack path, leading to the crack propagating in the middle of the adhesive layer.

### 3.3. Failure Strength and Extension of Epoxy Adhesive Joints with Respect to Bond Gap

**Figure 7:**
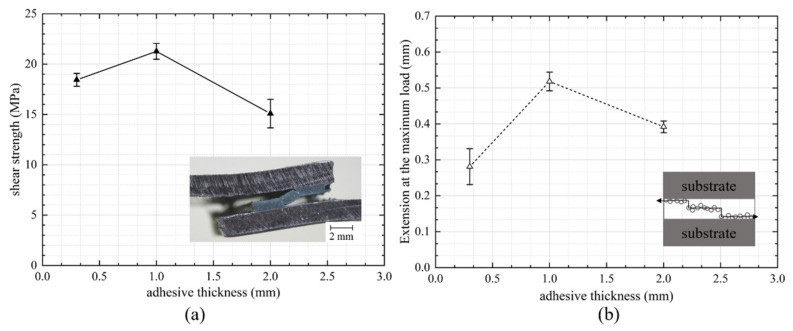
(**a**) Shear strength and (**b**) extension at the maximum load vs. thickness of the epoxy adhesive. The inset picture in the left indicates the bending of the aluminium substrates. The inserted right image shows the schematic drawing of the failure path within the adhesive.

Figure 7 shows the (a) failure strength and (b) extension at the maximum load as a function of adhesive thickness for the epoxy adhesive. Overall, less sensitivity to the bond gap was observed for the epoxy joints over the range studied. The failure strengths of the epoxy joints were much larger than the polyurethane joints (approximately six times for 0.3 mm bond thickness). Approximately 20 MPa was attained when the adhesive thickness was 1 mm, but it began to decrease when the adhesive thickness was larger than 1 mm. This can be explained by thicker bonds exhibiting more voids, microcracks, and other defects, which induced a greater probability of early failure [30,31]. It was also found that the joints experienced a large bending moment under high tensile load (Figure 7a), although alignment tabs were used. This bending moment was influential, inducing a higher peel stress at the overlap for thicker bond gaps and may have caused a sudden fracture. The extension at failure of the joints with different adhesive thicknesses also followed a similar trend (a decrease from around 1 mm to 2 mm). The maximum displacement of the 0.3 mm bond also presented a higher variation due to the unpredictable adhesive characteristics, as explained in Figure 4b. All the joints with epoxy adhesive failed in adhesion failure, in which cracks transferred from one interface to the other, as per the schematic shown in Figure 7b.

In general, the epoxy adhesives failed at a lower extension but at higher loads, and the metal substrates began to plastically deform. This led to a relatively small energy absorption capability. In contrast, the polyurethane adhesive could hold the joint together under higher deflection, and thus enabled larger energy absorption. Therefore, it is apparent that using various types of adhesives at different bond gaps is likely to have different impacts on the global performance of the vehicle structure. Whilst actual performance of the structure to failure is of interest to the vehicle designer, the actual joint stiffness will also play a key role in the vibrational performance of the vehicle.

### 3.4. Stiffness of the Joints

**Figure 8:**
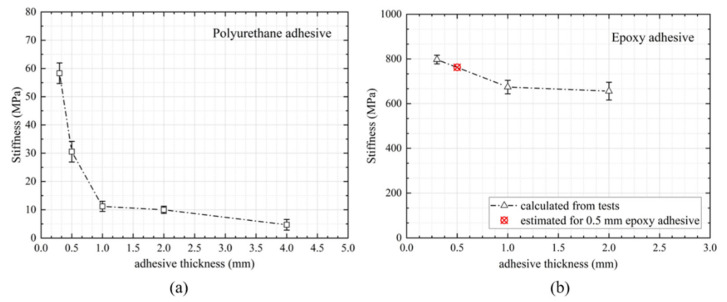
Stiffness of the joints vs. bond gap using (**a**) polyurethane and (**b**) epoxy adhesives. The point marked with a red cross in (**b**) is an interpolation of the joint stiffness for the 0.5 mm epoxy adhesive.

The stiffness of the joints bonded with both adhesives at different bond gaps are shown in Figure 8. The stiffness was calculated as the slope of the load-displacement line in the range from 5% to 10% of the maximum extension (elastic region), as it was considered that during this period, the adhesive was primarily bearing the shear load. Therefore, the calculated stiffness could be estimated as the joint shear stiffness regardless of the bending effect. The stiffness of the polyurethane joints dropped significantly with the increasing adhesive thicknesses (the stiffness dropped 90% when the thickness increased from 0.3 mm to 4.0 mm). For the epoxy adhesive, the joint achieved the highest stiffness with a thin bondline, and slightly reduced as the bond gap increased. In general, the epoxy adhesive was insensitive to the bond gap over the range studied. This could be expected, as at this initial stage with a smaller load, the joints were dominated by shear, and the shear deformation of epoxy adhesive did not vary much with the adhesive thickness compared to the polyurethane adhesive. As the joints approached failure, the bending moment of the joints became large and peel dominated; both peel and shear stresses tended to be higher for thicker epoxy adhesive, leading to a drop in the failure loads. It has been suggested that the stiffness drop of the joints was associated with the viscosity of the adhesive type and surface roughness of the joints [30]. The point marked with a red cross in Figure 8b is an interpolation of the joint stiffness for the 0.5 mm epoxy adhesive.

It should be noted that the stiffness values shown in Figure 8 corresponded to the shear stiffness of the joints with the substrates (as the 5% to 10% of the maximum extension was taken to reduce the bending moment effect), which were comparably larger than the adhesive’s shear modulus (0.9 MPa for polyurethane and 504 MPa for epoxy), as the substrate carried load as well. This shear stiffness was actually a common effect of the substrate and adhesive. However, compared to the substrate, the deformation of the adhesive was much larger than that of the substrate (the Young’s modulus of the substrate was almost 35 times that of the adhesive), thus the main deformation of the joint was mainly induced by the deformation of the adhesive.

Generally, the above studies were based on experimental studies for coupon-sized samples; it would also be interesting to discover how these behaviours affect the global performance of a rail structure with adhesive bonding as majority connections. A global vehicle model will be discussed in the next section to investigate the stiffness and modal behavior when using the different bonding scenarios, assuming that the bonding scenarios throughout the vehicles is simply single lap adhesive bonding.

## 4. Vehicle Finite Element Model

The FEM of the vehicle (Figure 1) was created using Hypermesh code and the OptiStruct solver. The vehicle model was built from the three-dimensional (3D) geometry produced by a design partner. A shell model was extracted by generating the midsurface from the 3D geometry to save computational cost/time. An element size of 10 mm was used after conducting a mesh convergence study. The entire steel bottom chassis was primarily joined by welding, in which RBAR elements were used in the model. For the joint modelling, the adhesive was modelled as a thin layer of shell elements with different thicknesses corresponding to the scenarios in the experimental tests and using simple tie constraints; assuming a perfect bond with the surrounding materials.

Therefore, the entire model has the following main characteristics:

Number of shell elements: 1,588,638

Number of structural grids: 1,620,384

The material properties of the main components are summarised in Table 1. The substrates and adhesives were modelled using an isotropic material card (MAT1). The composite material was modelled by an orthotropic material card (MAT8). The roof sandwich panel was modelled using a laminate section with different ply-based properties for the woven composite cover and phenolic foam core. The entire structure was expected to work under elastic conditions from modelling trials for conventional load states, so a linear elastic solver was an appropriate hypothesis. The performance of the vehicle was thus evaluated by adjusting the adhesive thicknesses according to the test results discussed earlier.

A torsional loading case was studied because it has been seen to result in the largest stresses in the vehicle body. The displacements and rotations were restricted at one end of the bogie mount connection point by rigid connections (RBE2 elements) to the surrounding nodes, while a total torsional load of 22,230 kN mm was applied to the other end as defined by the EN 12663 standard, which defines the structural requirements for railway vehicles [33]. Torsional stiffness is the characteristic property of a structure that signifies how rigid the structure is and how much resistance it offers per degree change in its angle when twisted, as shown in Equation (1). For calculating torsional stiffness:

(1) Ktorsion=Ttan−12utorsion/W

where *T* is the torsional load in N, *u*_torsion_ is the maximum deflection under torsional load in mm, and *W* is the distance between the centre of the two bogies, which is 7600 mm.

**Figure 9:**
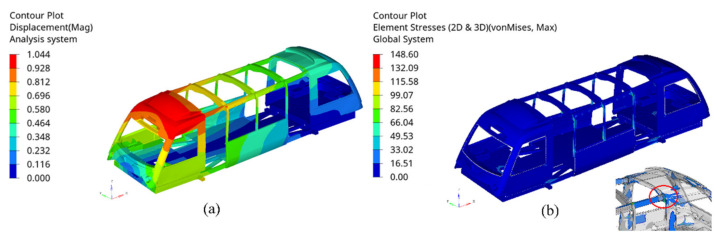
(**a**) Displacement and (**b**) stress distribution of the vehicle under torsional loading case using 0.3 mm epoxy adhesive (scale factor = 5). Units are in mm and MPa.

Figure 9 shows the (a) displacement and (b) stress distribution of the vehicle with the 0.3 mm thick epoxy adhesive under the torsional load. It can be seen that the vehicle exhibited a gradient deformation, with the largest distortion at the nose–roof joint section. The side module structures connected to the chassis sustained a high stress magnitude to resist large rotations, where the embedded adhesives also showed the highest stress value.

**Figure 10:**
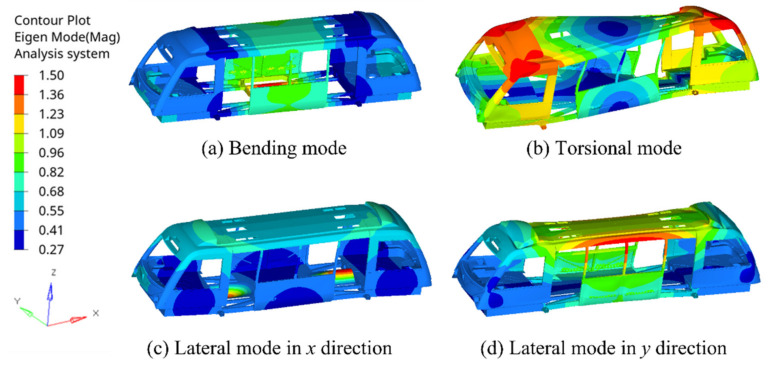
The most common mode shapes of the vehicle structure: (**a**) bending mode; (**b**) torsional mode; (**c**) lateral mode in x direction; and (**d**) lateral mode in y direction of the vehicle using 0.3 mm epoxy adhesive. The red zones indicate the structural components with high strain rates.

Further to torsional analysis, mode shapes and natural frequency have become an important evaluation parameter in vehicle applications to evaluate the necessity of design change, as they are dependent on the stiffness of the structures, and the mass participates within the structure. A modal analysis includes the frequency modes and natural frequency of a given system. In this paper, the normal modes analysis was carried out in the Hypermesh Optistruct package based on the Lanczos method [34], and the first three mode shapes and natural frequencies of the global behaviour were considered. The most common mode shapes of the vehicle structure appeared as (a) transverse (bending), (b) torsional, and (c,d) lateral modes, as shown in Figure 10.

## 5. Effect of Various Adhesives and Their Thicknesses on the Global Behavior

**Figure 11:**
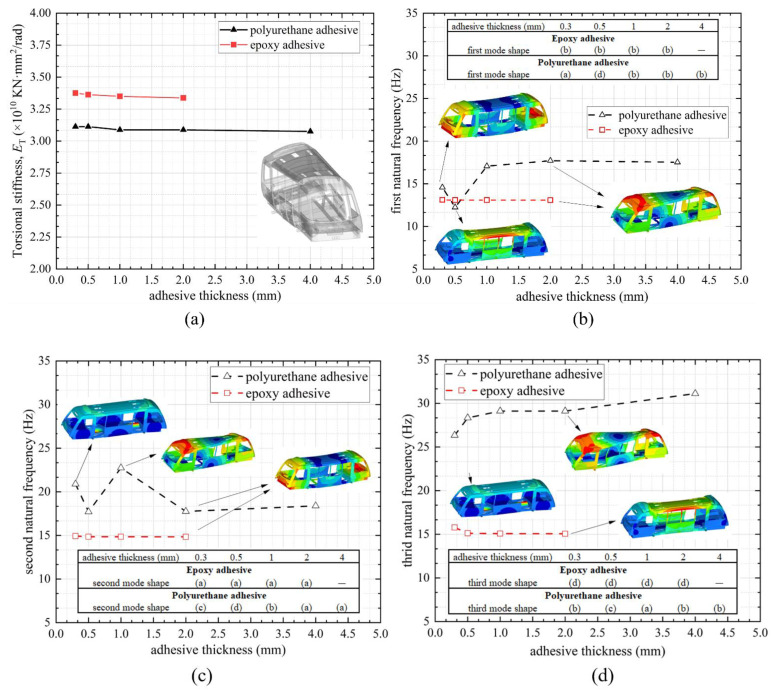
(**a**) Torsional stiffness, (**b**) first natural frequency, (**c**) second natural frequency, and (**d**) third natural frequency of the vehicle of different adhesive types and adhesive thicknesses. The colour legend shows the eigenvalue extraction, with the red colour indicating the maximum displacement (scale factor = 250). The model shapes refer to Figure 10.

As the adhesive thickness of the bonded assembly is difficult to control during manufacture, it is important to investigate the effect of the adhesive thickness on the vehicle’s global behaviour. FEM analysis was conducted at different adhesive thicknesses; Figure 11 indicates (a) torsional stiffness, (b) first natural frequency, (c) second natural frequency, and (d) third natural frequency of the vehicle using epoxy and polyurethane adhesives with different adhesive thicknesses. The vehicle with epoxy adhesive provided approximately 10% higher torsional stiffness compared to that with polyurethane adhesive, as the stiffness of the joint with epoxy adhesive was much larger, nearly 13 times for 0.3 mm thickness, as observed in the coupon tests shown in Figure 8. Interestingly, the torsional stiffness of the vehicle was insensitive to the adhesive thickness. It was assumed that as the torsional load was applied directly at the bogie mount structure that was welded to the lower chassis, the elastic deformations of the adhesive in terms of different thicknesses were relatively minor.

A significant variation was witnessed for the natural frequencies. Overall, the vehicle had a higher modal frequency value when using polyurethane adhesive compared to using epoxy adhesive. This was not unexpected, as the polyurethane adhesive was more flexible in large deformation and energy absorption, which can be witnessed in Figure 5. For the epoxy adhesive, the first natural frequency of the vehicle was approximately 13 Hz, and it was in torsional mode regardless of the adhesive thicknesses. This indicated that the top chassis and side module structure were stiffer than the nose assembly using structural adhesive (as the structural adhesive was mostly bonded to the roof assembly and side module skin), and the structural frequency remained similar even with higher adhesive thickness. The second and third natural frequencies of the vehicle were approximately 15 and 16 Hz, and in bending and lateral modes, respectively. Variation of the thickness of the epoxy adhesive had largely no effect on the vehicle’s first three natural frequencies.

For the polyurethane adhesive, the natural frequency of the vehicle varied more significantly with alterations in the thickness. The first natural frequency of the vehicle started from approximately 15 Hz in bending and torsional mode for 0.3 mm and 0.5 mm adhesive, respectively; nevertheless, as the thickness exceeded 1 mm, the mode peaked at approximately 17 Hz, and then it switched to a lateral mode. This implied that when using a thin polyurethane adhesive layer (less than 1 mm), the middle part of the vehicle was weaker, but it became able to perform a larger deformation and absorb more energy as the adhesive thickness increased, leading to an increased frequency and modal transformation. As the adhesive thickness increased beyond 2 mm, the first modal frequency further dropped due to the effect of a combination of the decreased joint stiffness and increased mass, which was successfully demonstrated in the coupon tests. The changes of the modal shapes could also be observed for the second natural frequency, from lateral mode (adhesive thickness smaller than 1 mm) to torsional (adhesive thickness of 1 mm) and to bending (adhesive thickness larger than 1 mm). This also occurred in the response of the third natural frequency. This indicated that the thickness of a low-shear-modulus adhesive played a significant role in altering the observed dynamic performance of the vehicle structure, and the peak natural frequency was achieved at a 1 mm adhesive thickness.

## 6. Conclusions

In this paper, experimental tests were reported from laboratory single lap joints with different types of structural adhesives (epoxy and polyurethane adhesives) and various adhesive thicknesses, with the aim of understanding the performance (shear strength and stiffness) of bonded joints at a coupon level. These different adhesive bonding strategies were then applied at a vehicle level to a state-of-the-art light rail structure with a multi-material design strategy to investigate the torsional stiffness and modal performance. Thus, it was shown that experimental data at a coupon level, combined with finite element analysis at a vehicle level, could be employed to assist engineering decision making for the viability and reliability of adhesive bonding in the rail industry. Based on the current study, the following conclusions could be drawn:

- For joints with polyurethane adhesive, the shear strength decreased significantly in terms of adhesive thickness. The joints with thick polyurethane adhesive experienced a relatively large shear deformation, leading to fracture and final failure. The shear strength of the joints with epoxy adhesive presented the highest value for a 1 mm bond, although the mechanical performance was far less sensitive to the bond gap compared to the polyurethane adhesive. At a high load, the aluminium substrates bonded with epoxy experience a large bending moment, leading to a concentrated peel stress at the interface, resulting in final fracture failure.
- The vehicle using epoxy adhesive behaved on average 10% stiffer than that using the polyurethane adhesive in sustaining torsional load; however, the torsional stiffness of the vehicle was largely not sensitive to the adhesive thickness; The vehicle with polyurethane adhesive had higher modal frequencies compared with that with epoxy adhesive, and the modal shapes also switched with increasing adhesive thickness.
- The polyurethane adhesive was more flexible and could bear large deformation, resulting in more energy absorption and a higher modal frequency. Therefore, the results indicated that an optimum thickness of approximately 1 mm of polyurethane adhesive is suggested for the current vehicle to optimise the structural performance. During the design phase of a vehicle, it is recommended that consideration be given to the effect of variation in fit-up during manufacturing, as this is likely to influence the vehicle’s vibrational response.

## Figures and Tables

**Table 1:** Properties of the materials used in the vehicle (material breakdown in Figure 1).

	Elastic Modulus (MPa)	Poisson’s Ratio	Density (kg/m^3^)
Steel Aluminium Woven CFRP ^a^	210,000	0.29	7850
	69,000	0.3	2700
	*E*_1_ = 76385 *E*_2_ = 69685	0.51	1400
Phenolic foam core Plywood	90	0.27	120
	4500	0.2	700
Sikaflex 265 adhesive [27]	2.7	0.48	1200
DP490 adhesive [28]	1442	0.38	1370

^a^ The material properties of the woven composites were tested and measured according to the ASTM D3039 standard [32].

**Figure A1:**
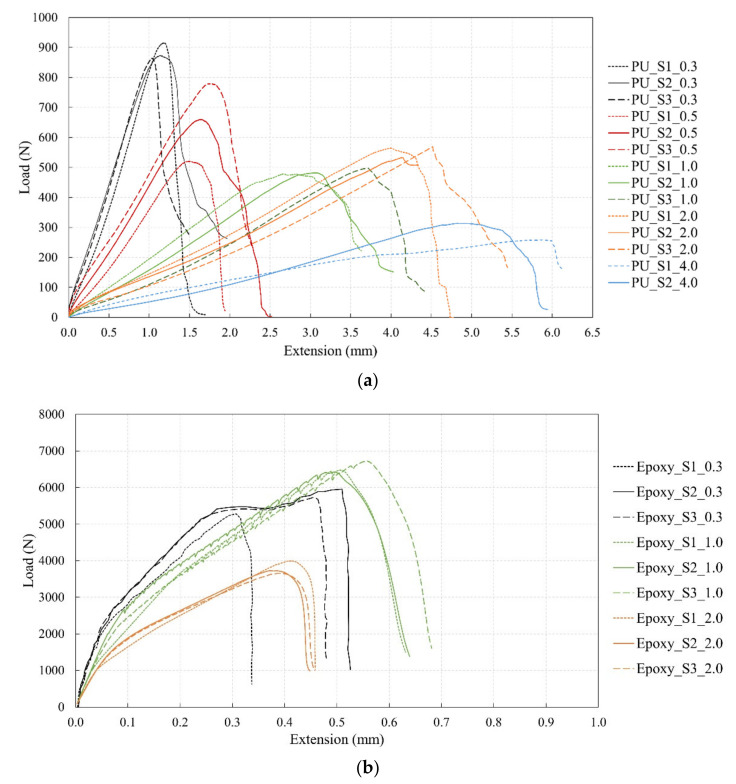
The load-extension curves of the adhesive bonded joints using (**a**) polyurethane adhesive (PU) with different adhesive thicknesses (0.3, 0.5, 1.0, 2.0 and 4.0 mm) and (**b**) epoxy adhesive with different adhesive thicknesses (0.3, 1.0 and 2.0 mm).

## Data Availability

Data sharing will be considered upon request.

## Appendix A

The load-extension curves of the adhesive bonded joints using polyurethane adhesive (PU) with different adhesive thicknesses (0.3, 0.5, 1.0, 2.0 and 4.0 mm) and epoxy adhesive with different adhesive thicknesses (0.3, 1.0 and 2.0 mm) are shown in Figure A1a,b in Appendix A, respectively. For the 4 mm PU adhesive, only two samples were used due to the difficulty in preparing samples.
